# Can a Gleason 6 or Less Microfocus of Prostate Cancer in One Biopsy and Prostate-Specific Antigen Level **<**10 ng/mL Be Defined as the Archetype of Low-Risk Prostate Disease?

**DOI:** 10.1155/2012/645146

**Published:** 2012-07-12

**Authors:** Gianluigi Taverna, Luigi Benecchi, Fabio Grizzi, Mauro Seveso, Guido Giusti, Alessandro Piccinelli, Alessio Benetti, Piergiuseppe Colombo, Francesco Minuti, Pierpaolo Graziotti

**Affiliations:** ^1^Department of Urology, IRCCS Istituto Clinico Humanitas, Rozzano, Milan, Italy; ^2^Department of Urology, Fidenza Hospital, Parma, Italy; ^3^Laboratories of Quantitative Medicine, IRCCS Istituto Clinico Humanitas, Rozzano, Milan, Italy; ^4^Department of Pathology, IRCCS Istituto Clinico Humanitas, Rozzano, Milan, Italy; ^5^Service of Statistics, IRCCS Istituto Clinico Humanitas, Rozzano, Milan, Italy

## Abstract

Prostate cancer (PC) remains a cause of death worldwide. Here we investigate whether a single microfocus of PC at the biopsy (graded as Gleason 6 or less, ≤5% occupancy) and the PSA <10 ng/mL can define the archetype of low-risk prostate disease. 4500 consecutive patients were enrolled. Among them, 134 patients with a single micro-focus of PC were followed up, and the parameters influencing the biochemical relapse (BR) were analysed. Out of 134 patients, 94 had clinically significant disease, specifically in 74.26% of the patients with PSA <10 ng/mL. Positive surgical margins and the extracapsular invasion were found in 29.1% and 51.4% patients, respectively. BR was observed in 29.6% of the patients. Cox regression evidenced a correlation between the BR and Gleason grade at the retropubic radical prostatectomy (RRP), capsular invasion, and the presence of positive surgical margins. Multivariate regression analysis showed a statistically significant correlation between the presence of surgical margins at the RRP and BR. Considering a single micro-focus of PC at the biopsy and PSA serum level <10 ng/mL, clinically significant disease was found in 74.26% patients and only positive surgical margins are useful for predicting the BR.

## 1. Introduction

Prostate cancer (PC) remains the most common cancer in men, and its prevalence in men aged >50 years has been estimated to be as high as 40% [1]. PC still represents the third leading cause of male cancer-related death, after lung and colorectal cancer [2], but the majority of cases are nonlethal [3]. Although it is true that radical treatment significantly decreases the risk of death from PC, it is also true that 19 men need to be treated to benefit one man [4, 5]. This arises from the prostate-specific antigen (PSA) screening Era, although the helpfulness of PSA screening still remains debated.

Andriole et al. report no mortality benefit from combined screening with PSA testing and digital rectal examination (DRE) during a median follow up of 11 years [6], while Schröder et al. [7] report that PSA screening without DRE is associated with a 20% relative reduction in the death rate from PC at a median followup of 9 years.

However, it remains indubitable that the combined use of PSA and transrectal ultrasound-guided needle biopsy as screening procedure has diagnosed an increasing number of PC, overall at an earlier stage (i.e., low PSA value, grade, and tumour volume) [8]. 

This has generated several doubts on the risks of overdiagnosis and overtreatment of insignificant neoplastic diseases. It is unclear whether all patients diagnosed with PC warrant radical treatment or may benefit from delayed intervention following active surveillance. The challenge remains to distinguish accurately those potentially dangerous lesions from nontreating cancers.

The above considerations have led to categorize patients with PC in three groups with low or insignificant, intermediate, and high risk.

During the last years, different definitions of insignificant or low-risk PC have been proposed [9]. However, all of them have highlighted limits in patient stratification. Using the more restrictive definition, low-risk PC might be defined as that detected in patients with PSA <10 ng/mL, stable PSA kinetics, Gleason grade ≤6, and Clinical Stage T1/T2a [10]. It is known that patients with low-risk cancer have 10-year PC survival rates in excess of 99% [10, 11], while is still uncertain whether intervention improves a longer survival time. To avoid that patients will undergo radical treatment for presumed clinically insignificant PC, active surveillance has been applied as an alternative option to an immediate treatment [9–16]. According to Stamey et al., a tumour with a volume <0.5 mL and a Gleason score <7 would not be life threatening, because such PC have a long doubling time [16]. On the basis of these premises in the category of low-risk PC, a sub-group of patients with a diagnosis of a single microfocus (defined as ≤5% occupancy in 1 biopsy core with Gleason grade ≤6), PSA <10 ng/mL and clinical stage T1c, represents the archetype of low-risk PC. Despite various definitions of “cancerous microfocus,” have been proposed, the risk of finding clinically insignificant disease at successive RRP varies from about 9% to 40% [17–20]. 

In addition, Thong et al. have found an association between the presence of a single microfocus at biopsy and BR after robotic prostatectomy in about 3% of patients [21].

The above considerations raise the following questions: are the actual parameters for predicting the real biological impact of the disease really helpful? And, is the pathological definition of insignificant PC disease still valid? [22–25].

Here we correlated the detection of a tumoural microfocus at even repeat prostatic biopsy and the presence of clinically significant disease detected after RRP and patient's followup. The definition of clinically significant disease has been reevaluated in the light of the biochemical relapse (BR). Additionally, we verified whether some preoperative and pathological parameters could be helpful in identifying subgroups of patients who may need more or less aggressive and/or timely appropriate treatment.

## 2. Materials and Methods

### 2.1. Patients

4500 consecutive patients who underwent sextant prostate biopsies at the IRCCS Istituto Clinico Humanitas, Rozzano, Milan, Italy and at the Fidenza Hospital, Parma, Italy between January 2000 and September 2008 were enrolled in the study. All of them had high preoperative PSA levels (>2.5 ng/mL) and/or abnormal results upon digital rectal exploration (DRE). 

Their PSA density (ultrasonography PSAD) and *f*/*t* ratios were tested and monitored overtime, and the volume of their prostate glands was estimated ultrasonographically using the elliptical method [26]. None of the patients had received neoadjuvant therapy or treatment with 5-alpha reductase inhibitors. All of the patients were clinically followed (including DRE and PSA determinations) every six months. BR was defined as detectable PSA level (>0.2 ng/mL) with increase of this value during the followup. All of the patients underwent ultrasound-guided modified sextant prostatic needle biopsies under local anaesthesia induced by a single dose of lidocaine [25]. The mean number of biopsy cores per procedure was 13 (range: 11–20 biopsy cores), and they always included the transitional zone, apex, and lateral part, as well as the midmedial, midlateral, basal, and anterior zones [27].

### 2.2. Histological Analysis

Radical retropubic prostatectomy (RRP) specimens were weighed, fixed in 10% formalin, embedded in paraffin, serially cut at 3 *μ*m intervals, and subsequently histochemically stained with a freshly made haematoxylin and eosin solution for the microscopy observation by the same uropathologist (PC). For each patient anatomo-clinical parameters, including the percentage of neoplastic disease, Gleason score, capsular invasion, and surgical margins, were evaluated.

A microfocus of prostate adenocarcinoma was defined as ≤5% occupancy of 1 biopsy with Gleason grade ≤6 [22, 23, 26, 27]. Patients with Gleason grade >7, and/or with tumour foci in more than one biopsy core, as well as those with a diagnosis of high-grade prostatic intraepithelial neoplasm (PIN) or atypical small acinar proliferation (ASAP) were excluded by the study.

Clinically insignificant prostate carcinoma was defined as the presence of a tumour in 5% of the glands with a Gleason grade <7 [17, 24]. 

### 2.3. Statistical Analysis

Categorical data are presented as absolute frequency and percentage proportion; continuous data are presented as median and range. Parametric and nonparametric tests were used as appropriate in order to evaluate the significance of differences in variables' distribution (*t*-test, Fisher's exact test, Wilcoxon's rank-sum test, and Pearson's *χ* ^2^). Survival analysis was carried out according to the Kaplan-Meier method and the Cox proportional hazards regression model to evaluate prognostic factors related to the biochemical relapse. A significance level of 5% was adopted.

## 3. Results 

The needle biopsies showed that 194 of 4500 (4.3%) patients had a single microfocus of adenocarcinoma. 134 out of 194 (67%) patients who underwent RRP nerve sparing (PSA 6.3 ng/mL (range: 2.1–33 ng/mL), *f*/*t* ratio 15% (range: 0.5–44%), and PSAD 0.12 (range: 0.05-06) were adequately informed about the available diagnostic and therapeutic possibilities, as well as the published criteria regarding the definition of clinically insignificant disease. 11 out of 134 (8%) patients were to undergo radiotherapy and 49 out of 194 (25%) were to undergo active surveillance. 94 out of 134 patients (70.15%) were diagnosed as affected by clinical significant disease, while 40 (29.85%) patients had clinical insignificant disease at the successive RRP.

101 out of 134 patients (75.37%) had PSA <10 ng/mL. In 75 (74.26%) patients a clinically significant disease was found, while in 26 (25.74%) a clinically nonsignificant disease was detected at the successive radical prostatectomy.

33 (24.63%) patients had PSA >10 ng/mL. In 19 (57.58%) patients a clinically significant disease was found, while in 14 (42.42%) patients a clinically non-significant disease at the successive radical prostatectomy was detected. No statistically significant differences were found between the percentages of clinically significant disease (*P* = 0.069) and *f*/*t* ratio (*P* = 0.98). In contrast, a higher PSAD was found in patients with PSA >10 ng/mL (*P* ≤ 0.001).

39 (29.1%) patients showed positive surgical margins. In 34 out of 101 (33.66%) patients with PSA <10 ng/mL had positive surgical margins, while 5 (15.15%) patients with PSA >10 ng/mL had positive surgical margins. Incidentally, the percentage of positive surgical margins was found statistically significant in the group of patients with PSA <10 ng/mL (*P* = 0.042). 

69 out of 134 (51.49%) patients had capsular invasion. In 54/101 (53.47%) patients with PSA <10 ng/mL were found capsular invasion. In 15/33 (45.45%) of patients with PSA >10 ng/mL had capsular invasion. No statistically significant differences were found between two groups with regard to capsular invasion at the successive RRP (*P* = 0.424).

The following pathological stages have been scored: 2 (1.49%) pT0; 32 (23.88%) pT2aN0; 28 (20.90%) pT2bN0; 47 (35.07%) pT2cN0; 20 (14.93%) pT3aN0 and 5 (3.73%) pT3bN0. Classifying the patients on the basis of the PSA behaviour, no statistically significant differences have been found, although a prevalence of the stage pT2cN0. No statistically significant differences have also been found between patients with a Gleason score ≤6 and those ≥7 (*P* = 0.202).

We divided the patients to two groups: 87 (64.93%) of them underwent RRP after a first needle biopsy with a microfocus of adenocarcinoma *(group 1)* and 47 patients (35.07%) underwent RRP after a first biopsy had shown a microfocus of adenocarcinoma and a second had led to the identification of a tumour >5% occupancy of 1 biopsy with Gleason grade >6, or with multiple adenocarcinoma foci *(group 2)*. 

*Group 1* included 87 (64.93%) patients with a mean PSA equal to 7 ng/mL (range: 2.6–33 ng/mL); *f*/*t* ratio 13.05 (range: 4–44); PSAD 0.14 (range: 0.046–0.6). In 65 (74.71%) of them a clinically significant disease has been found, while in 22 patients (25.29%) the disease was clinically insignificant.

*Group 2* included 47 (35.07%) patients with a mean PSA equal to 6.1 ng/mL (range: 2.6–25 ng/mL), *f*/*t* ratio 17.5 (range: 0.5–40), and PSAD 0.12 (range: 0.051–0.384). In 29 patients (61.70%) a clinically significant disease has been found, while in 18 (38.30%) patients the disease was clinically insignificant.

There were no statistically significant differences between two groups with respect to the percentage of clinically significant disease (*P* = 0.116), PSA levels (*P* = 0.407), and *f*/*t* ratio (*P* = 0.169). PSAD was higher in *Group 1* (*P* = 0.013).

Additionally, there were no statistically significant differences between two groups with respect to the Gleason grade and pathological stage, although two patients with stage pT0 belong to *group 1*. 

We analysed the percentage of BR after patient followup considering all the clinical, biochemical and pathological stage to highlight any positive prognostic factors. 

118 out of 134 (88.06%) patients have been analysed. 2 patients died during the followup for other causes (1 colorectal cancer and 1 pancreatic cancer), while 14 patients were lost. The median followup was 28 months (range: 3.7–96 months). 35 out of 118 (29.6%) patients had BR.

Using the Cox regression, the following parameters were found statistically significant with the biochemical relapse: Gleason score (HR = 2.94, *P* = 0.009), the capsular invasion (HR = 3.56, *P* = 0.006), and positive surgical margins (HR = 6, *P* < 0.001). 

In contrast, there was no statistical significance between BR and PSA value (*P* = 0.974), pathological stage (*P* = 0.168), definition of clinically significant disease (*P* = 0.070), confirm at rebiopsy (*Group 1* versus *Group 2*) (*P* = 0.860), *f*/*t* ratio (*P* = 0.625), and PSAD (*P* = 0.318). We therefore, evaluated the effect of significant variables taken together (multivariate Cox regression) and found that only the presence of positive surgical margins is a predictive factor for the BR (*P* = 0.006).

## 4. Discussion

Approximately 32% of male Caucasians aged more than 50 years have PC at autopsy. The lifetime risk of developing PC in the United States is 1 in 6, and the lifetime risk of death due to metastatic PC is 1 in 30 [28]. The advent of PSA testing has changed our understanding of the natural history of the prostatic disease [16–30], but the widespread use of repeated and extended biopsies has increased the rate of patients with apparently clinically insignificant disease. This problem, in epidemiologic term, is defined as the diagnosis of cancer that will not be diagnosed clinically during life [31].

The above considerations demonstrate the difficulty to correctly select the patients to be treated (i.e., radical prostatectomy, radio-therapy, and active surveillance) and how to define the criteria of risk of progression of the neoplastic disease.

Within the low-risk group there is a subgroup of patients with a single microfocus of PC at biopsy with PSA <10 ng/mL which, according to the existing parameters, represents the archetype of the disease at low-risk and therefore less than that should provide for a radical treatment. 

Current data on the proportion of clinically significant disease in the presence of a microfocus remain contradictory, and the risk of finding clinically insignificant disease varies from about 9% to 40% [17–23, 26–33].

In our retrospective study, we selected 134 patients who underwent nerve-sparing RRP after the histological diagnosis of a single microfocus of PC, associated or not with a second confirmation of disease at repeat biopsy. Clinically significant disease was found in 70.15% patients and more specifically in 74.26% of patients with PSA <10 ng/mL and 57.58% in those with PSA >10 ng/mL (*P* = 0.069). 

We examined several clinical and epidemiological parameters including clinical stage, *f*/*t* ratio, PSAD, and age, and pathological parameters such as surgical margins, Gleason grade, pathological stage, and capsular invasion, to assess whether the group with the lower-risk disease corresponded to a minor impact. Surprisingly, we found a statistically significant difference between the group with PSA levels <10 ng/mL and that with PSA levels >10 ng/mL with respect to positive margins being higher in the group with PSA <10 ng/mL. With regard to capsular invasion, the pathological stage and Gleason score there were not observed statistically significant differences between the two groups. 

We then analysed in 35% of patients the possible impact of histological confirmation at rebiopsy although we found that it did give not additional information and did not reduce the risk of clinically significant disease as confirmed at the subsequent RRP. 

Finally, we evaluated the followup of these patients and have found that none of the patients died from PC. Although the period of observation is limited as much as 29.6% of patients showed a BR. Using the Cox regression we found a statistical significance among the BR and the Gleason score (HR = 2.94, *P* = 0.009), the capsular invasion (HR = 3.56, *P* = 0.006), and the presence of positive surgical margins (HR = 6, *P* < 0.001). In contrast, there was no statistical significance with PSA level, pathological stage, definition of clinically significant disease, confirm at rebiopsy, *f*/*t* ratio, and PSAD.

Then, when we examined the multivariate Cox regression we found that only the presence of positive surgical margins appears to be the factor related to the BR. On the basis of the above results emerges that the predictive values we investigate have not proved helpful to carefully selected patients.

There is an urgent need to understand the significance of insignificant PC [24]. In a study by Allan et al. 54 patients were identified with a Gleason grade 6 single microfocus of PC on prostate needle biopsy [22]. While twothirds of the patients had potentially insignificant tumours at radical prostatectomy, a third of their cohort harboured clinically significant tumours exceeding the actual criteria (i.e., Gleason score ≤6, organ confined, and a volume <0.5 cm^3^). Allan et al. also reported that a PSAD <0.15 ng/mL correlated with potentially insignificant tumours at the RRP [22]. In our study, the PSAD, obtained by ultrasonography, it has not proved useful to select patients because the relationship with BR is mainly due to the presence of positive margins and thus the seat of the tumour and pathological stage or size of the prostate for equal PSA levels. 

In a more recent study similar findings correspond to that observation since 22% of the cases were upstaged to pT3 and/or upgraded to Gleason score 7 or greater [21]. In our study 74% of the patients showed at the RRP a clinically significant PC (pT2b + pT2c + pT3a + pT3b).

The real problem is the excess of confidence on the staging possibilities of extensive prostate biopsy. The primary outcome of the prostate biopsy sampling for diagnosing PC is the increase of the number of biopsy cores to increase the diagnostic sensitivity. The biopsy has a quality value. The turn in a prognostic value of a sampling percentage is not immediate.

In fact in a cohort studied by Sheridan et al. men with low-risk prostate cancer, defined as Gleason grade ≤6, fewer than 3 positive cores with no cores occupied by greater than 50% of cancer and clinical PSAD less than 0.15, who were undergoing active surveillance were at 19% risk of upgrading on subsequent biopsies [34]. Most tumours were upgraded within 24 months of the initial diagnosis, suggesting that a higher-grade tumour was not sampled in the original biopsy. Observations in the study of Thong [21] support these findings because 18% of cases were upgraded at surgery. Therefore needle biopsy alone is not sufficiently reliably to differentiate tumours that are clinically insignificant and those that are upstaged or upgraded to clinically significant tumours, which more clearly warrant definitive treatment.

Likely to the PSA we have not shown statistically significant differences in the proportion of clinically significant disease among the group of patients with PSA <10 ng/mL and those with PSA >10 ng/mL. Moreover, the fact that the group of patients with PSA >10 ng/mL, that is, theoretically with a more aggressive disease showed a lower percentage of positive surgical margins compared to the group of patients with PSA <10 ng/mL. These results all confirm the low predictive sensitivity of PSA in the patient groups analysed. Although the claimed importance of rebiopsy [20], we here have not shown statistically significant differences regarding the proportion of recognized clinically significant disease, the Gleason score, and pathological stage disease when compared to patients not undergoing rebiopsy, and more importantly between the two groups with respect to BR.

Regarding the resumption of BR we have observed that this was related to the Gleason grade, capsular invasion, and positive surgical margins. But by performing a multivariate Cox regression only the positive surgical margins were related to BR.

Importantly in all cases investigated in our study, in addition to that PSA and PSAD were not indicative nor the definition of disease is not clinically significant, which is based on the rate of disease and not on the premises. Smaller cancers but closer to the margin is more risky for the resumption of the disease. Thong et al. have reported that 3% of recovery biochemical disease in a group of patients undergoing robotic RP after detection of a microfocus at biopsy [21]. 

The percentage of biochemical relapse appears lower than that found in our study (3% versus 29.6%).

In comparison to the study of Thong et al. the percentage of positive surgical margins is unquestionably more elevated. By analysing only the patients staged as pT2, we detected a percentage of 19.6% of cases with positive margins. Although the value remains high a variable range comprises between 2% and 49% has been reported [35]. Comparing our data with those reported by Ojea Calvo et al. and other authors [35], we found a percentage of positive surgical margins equal to 9.3% versus 6.9 for pT2a cases and 28.5% versus 18.6% for pT2b cases. In literature it emerges that today it is not possible to foresee result with certainty and therefore to avoid to produce positive margins [35]. Therefore the difference with the percentages published by Thong et al. can mainly result from the population in examination, the histopathological examination, or from the actual surgical procedures. With regard to the BR, our results agree to those of Gardner et al. that underline, with a followup of 24 months, the 6% of resumption biochemistry for microfocus with 6% of positive margins [17]. This difference can be justified on the basis of the shorter followup (12.2 months, range: 1.2–53 versus 28 months, range: 3.7–96 months), or to the percentage of positive margins (6% versus 29.1%). It should be underlined that a limit of our study consists, although great in comparison to other published studies, in the time of followup.

## 5. Conclusions

We can conclude that there exists a nonclinically significant disease; it is indubitable that we know an overtreatment of an unnecessarily large group of patients, but this is mainly due to the scarce knowledge on the complexity underlying the progression of the tumoral prostatic disease, the multiscale causality that is intrinsically determining the dynamical behaviour of PC, and the fact that actual parameters do not recognize the risk of disease progression and are incapable to accurately select patients to be treated. Our results show that, to date, the predictive value that we look in hope to select patients may even be misleading; making choice of treatment over another lead to error, and therefore only the introduction and development of new and different predictive values can overcome this impasse. While remaining clear the axiom that only a small fraction of patients with PC dies because of disease and act on all over would entail an unacceptable treatment, and we are not yet able to identify with certainty those who may simply be observed. From our experience, in fact, the detection of a single microfocus in one biopsy with Gleason grade ≤6 and PSA <10 ng/mL cannot be identified as a sufficient parameter to define an indolent disease.
